# Exploring patients' perspectives of gestational diabetes mellitus screening and counselling in Ontario: A grounded theory study

**DOI:** 10.1111/hex.13708

**Published:** 2023-01-18

**Authors:** Emma Ruby, Sarah D. McDonald, Howard Berger, Nir Melamed, Jenifer Li, Elizabeth K. Darling, Jon Barrett, Joel G. Ray, Michael Geary, Beth Murray‐Davis, DOH‐NET (Diabetes, Obesity and Hypertension in Pregnancy Research) and SOON (Southern Ontario Obstetrical Network) Investigators™

**Affiliations:** ^1^ Department of Obstetrics and Gynecology, McMaster Midwifery Research Centre McMaster University Hamilton Ontario Canada; ^2^ Departments of Obstetrics and Gynecology Radiology and Clinical Epidemiology and Biostatistics, Division of Maternal‐Fetal Medicine McMaster University Hamilton Ontario Canada; ^3^ Department of Obstetrics and Gynecology, Division of Maternal‐Fetal Medicine, St. Michael's Hospital University of Toronto Toronto Ontario Canada; ^4^ Department of Obstetrics and Gynecology, Division of Maternal‐Fetal Medicine, Sunnybrook Health Sciences Centre University of Toronto Toronto Ontario Canada; ^5^ Department of Obstetrics & Gynaecology Rotunda Hospital Dublin Ireland

**Keywords:** counselling, gestational diabetes, grounded theory, midwifery, obstetrics, screening

## Abstract

**Introduction:**

Gestational diabetes mellitus (GDM) is associated with adverse perinatal outcomes. Approaches to screening for GDM continue to evolve, introducing potential variability of care. This study explored the impact of these variations on GDM counselling and screening from the perspectives of pregnant individuals.

**Methods:**

Following a Corbin and Strauss approach to qualitative, grounded theory we recruited 28 individuals from three cities in Ontario, Canada who had a singleton pregnancy under the care of either a midwife, family physician or obstetrician. Convenience and purposive sampling techniques were used. Semi‐structured telephone interviews were conducted and transcribed verbatim between March and December 2020. Transcripts were analysed inductively resulting in codes, categories and themes.

**Results:**

Three themes were derived from the data about GDM screening and counselling: ‘informing oneself’, ‘deciding’ and ‘screening’. All participants, regardless of geographical region, or antenatal care provider, moved through these three steps during the GDM counselling and screening process. Differences in counselling approaches between pregnancy care providers were noted throughout the ‘informing’ and ‘deciding’ stages of care. Factors influencing these differences included communication, healthcare autonomy and patient motivation to engage with health services. No differences were noted within care provider groups across the three geographic regions. Participant experiences of GDM screening were influenced by logistical challenges and personal preferences towards testing.

**Conclusion:**

Informing oneself about GDM may be a crucial step for facilitating decision‐making and screening uptake, with an emphasis on information provision to facilitate patient autonomy and motivation.

**Patient or Public Contribution:**

Participants of our study included patients and service users. Participants were actively involved in the study design due to the qualitative, patient‐centred nature of the research methods employed. Analysis of results was structured according to the emergent themes of the data which were grounded in patient perspectives and experiences.

## 1 BACKGROUND

Gestational diabetes mellitus (GDM) is one of the most frequent metabolic disturbances of pregnancy, affecting up to 20% of pregnant individuals in Canada.^1^ Factors contributing to the rising rates of GDM in Ontario include variations in screening approaches and diagnostic thresholds, and increased incidence of delayed childbearing, obesity, and excess gestational weight gain.^2^, ^3^, ^4^, ^5^

Two approaches to GDM screening are endorsed by Diabetes Canada (DC) and the Society of Obstetricians and Gynecologists of Canada (SOGC): the ‘preferred’ two‐step, method of a nonfasting, 50 g oral glucose challenge test (50 g OGCT) followed by a fasting 75 g oral glucose tolerance test (75 g OGTT) upon abnormal results, as well as an ‘alternate’ method of the one‐step, fasting 75 g OGTT.^6^, ^7^, ^8^ A randomized controlled trial, comparing the incidence of GDM between screening approaches, revealed a nearly doubled incidence rate of GDM in the group who underwent a one‐step 75 g OGTT, when compared to the group who was administered a 50 g OGCT followed by a 100 g OGTT (16.5% vs. 8.5%, respectively).^9^

However there is a lack of consensus between regulatory bodies on the optimal GDM screening approach. For example, DC and the SOGC recommendations differ from that of the International Association of the Diabetes and Pregnancy Study Groups (IADPSG), which supports the use of the one‐step 75 g OGTT with lower diagnostic cut‐off values.^7^, ^8^, ^10^, ^11^ Comparatively, the American College of Obstetricians and Gynecologists (ACOG) recommends the use of an OGTT with a higher glucose load (100 g) and a longer assessment period (3 h).^11^, ^12^ A recent randomized controlled trial examined the rate of GDM diagnosis across groups with different diagnostic threshold criteria, with the lower glycemic criteria group reporting over double the rate of GDM diagnoses compared to the higher glycemic criteria group (15.3% vs. 6.1%, respectively).^13^

There has also been debate over universal versus risk‐based GDM screening. The SOGC and DC shifted from selective screening in the late 1990s to recommending universal screening.^6^ Differences and uncertainty of diagnostic thresholds between approaches, the most appropriate glucose load, the number of abnormal values required to determine a GDM diagnosis, the importance of early trimester and postpartum screening, as well as whether to practice risk‐based or universal screening, has led to discrepancies in the true prevalence of GDM in Canada.^6^

From the patient's perspective, GDM care includes a range of experiences such as counselling, screening, diagnosis, management and postpartum follow‐up. Patients who received a GDM diagnosis have reported feelings of self‐blame, failure, confusion, and fear, signifying uncertainty and guilt.^14^, ^15^, ^16^ Lack of time and continuity of care have been identified as barriers to communication between patients and healthcare providers (HCPs); however, discussion of the GDM condition, associated risks and potential outcomes have been shown to promote greater acceptance of the diagnosis for the patient.^14^

Despite evidence indicating patient motivation to protect the health of their baby, many challenges impeding positive behaviour change and treatment compliance have been reported.^17^ These have included a lack of access to GDM services, financial barriers, lack of communication with HCPs and poor adherence to lifestyle changes such as diet and exercise.^17^ Given the multifaceted nature of GDM for patients, the aim of this study was to explore the impact of variations in GDM counselling and screening from the perspective of patients.

## 2 METHODS

We conducted a qualitative, grounded theory study with pregnant participants to explore their experiences of gestational diabetes screening and counselling practices. We sought to recruit participants from various geographic locations in the province, and who received care from different antenatal care providers. Patients who had had a singleton birth within the 5 months before data collection, who also received antenatal care from a midwife (MW), family physician (FP) or obstetrician (OB) within Hamilton, Ottawa or Sudbury, Ontario, Canada, were eligible to participate. Semi‐structured interviews took place between March 2020 and December 2020 and were conducted over the telephone for approximately 30–45 min. Convenience and purposive sampling were used for recruitment, using social media and posters within the community. These sampling methods were chosen based on the limited geographic regions targeted and the ability to identify participants with lived experiences with the research topic. Additionally, given the lack of strict selection criteria and the qualitative methodology employed, these sampling techniques were most appropriate.

The geographic regions identified for inclusion were selected to increase subpopulation variability and to utilize existing contacts to assist in recruitment. A minimum of three patients who received care from each health profession and from each geographic region was identified as the desired sample size (totalling 27 participants) based on experience with similar studies by the research team, but with the intention of continuing recruitment until we reached saturation.^18^, ^19^ An interview guide was developed by our research team and utilized during the interview process, using a mix of open‐ and closed‐ended questions to elicit the participant's perspectives on their experiences with GDM counselling and screening (see Supporting Information: S1).

In keeping with grounded theory, as described by Corbin and Strauss, data analysis began at the same time as data collection, to make use of the iterative process of constant comparison.^20^, ^21^ Interviews were transcribed verbatim and entered into Nvivo 11 software.^22^ Data analysis began with open coding. Initial open coding of three transcripts was completed by three independent researchers to ensure consistency and agreement in the coding process.^23^ Next, codes were grouped to form axial codes which provided a framework from which the open codes could be synthesized into hierarchically structured categories.^24^, ^25^, ^26^, ^27^ Lastly, during the selective coding process, further grouping was completed to form themes that, when brought together, generated a theory grounded in the data.^27^

Interim analyses were shared at team debrief meetings, and the Principal Investigator reviewed the coding at each stage of analysis.^23^ The research team was comprised of students and experts from a variety of disciplines, including midwifery, maternal‐foetal medicine, obstetrics and health research methodology. Investigator triangulation was used to review, validate and come to an agreement on disputed codes between researchers. These approaches were employed to minimize bias, strengthen credibility and add breadth to the emerging phenomena.^28^

## 3 RESULTS

A total of 28 participants were included. Demographic characteristics were obtained and are presented below (Table 1).

**Table 1:** Participant characteristics by the antenatal care provider

Characteristics	Antenatal care provider			
	Midwife (*n* = 12)	Obstetrician (*n* = 10)	Family physician (*n* = 6)	Total (*n* = 23^a^)
Geographic region				
Hamilton	5	3	2	10
Ottawa	4	4	3	11
Sudbury	3	3	1	7
Maternal age (years)				
15–24	0	0	1	1
25–34	6	6	1	13
35–44	3	4	2	9
Ethnic or cultural origin(s)				
East Asian	0	2	0	2
White	9	8	4	21
Hispanic	0	1	0	1
Other	0	0	0	0
Highest level of education				
High school	0	2	1	3
Bachelor's degree	4	5	2	11
Master's degree	4	1	1	6
Postgraduate certificate	0	1	0	1
Doctorate	1	0	0	1
College diploma	0	1	0	1
Past live births, including most recent (#)				
1	6	8	3	17
2	3	1	1	5
3	0	1	0	1
BMI (kg/m^2^)				
18–24	5	4	4	13
25–30	4	0	0	4
30–35	0	2	0	2
35–40	0	3	0	3
>40	0	1	0	1
Neonatal birth weight (lbs)				
<6	1	1	1	3
6–7	3	1	1	5
7–8	2	5	1	8
8–9	2	2	1	5
>9	1	1	0	2
Gestational age at delivery (weeks + # days)				
<35	1	0	0	1
35–37 + 6	1	2	1	4
38–39 + 6	2	7	0	9
≥40	4	1	3	8
Diagnosis of GDM in most recent pregnancy (Yes/No)				
Yes	2	1	0	3
No	7	9	4	20

^a^ Demographic characteristics data from five participants were not obtained.

**Figure 1:**
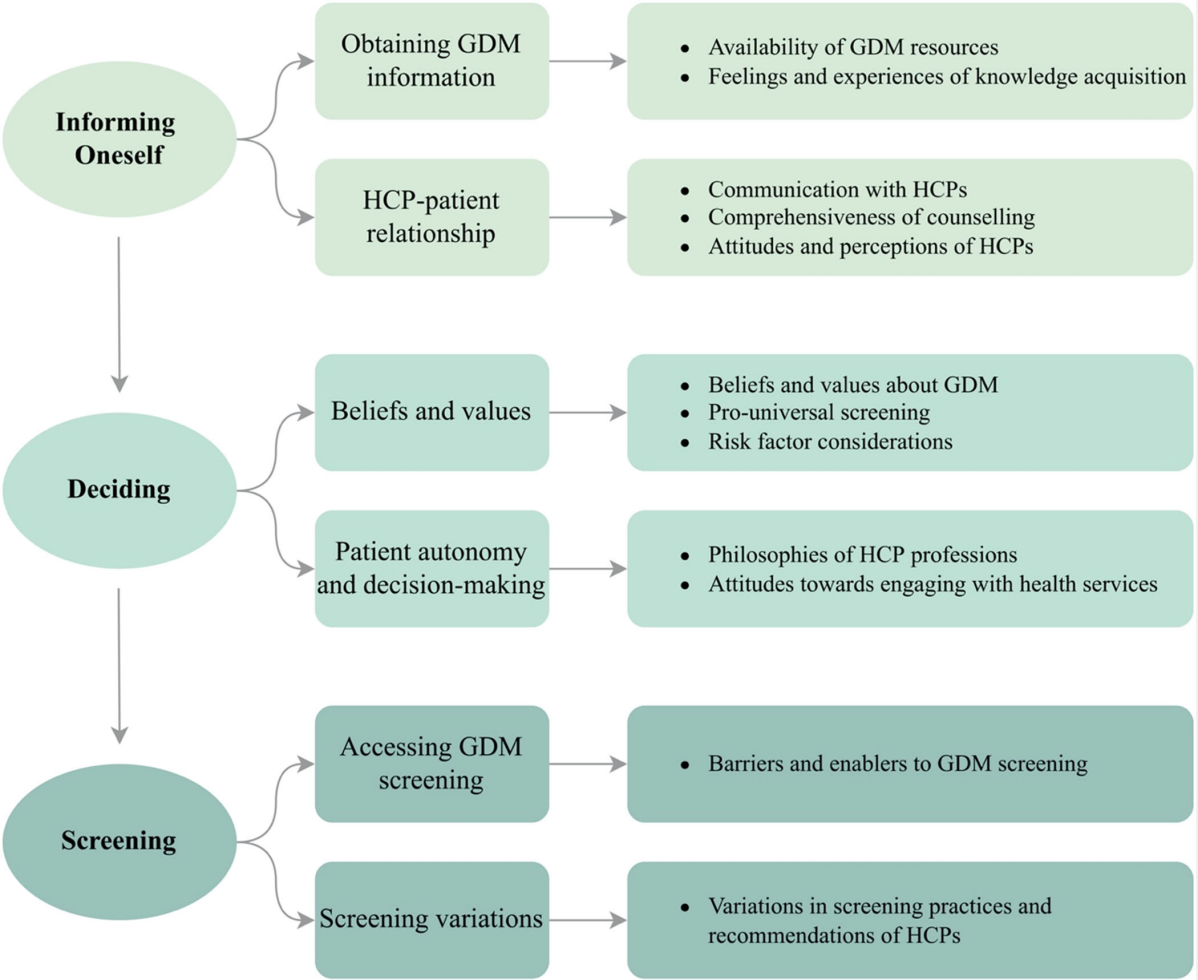
Categorization of participants' experiences with GDM counselling and screening practices. GDM, gestational diabetes mellitus; HCP, healthcare provider.

Our findings are summarized in the themes arising from the data: ‘informing oneself’, ‘deciding’ and ‘screening’ (Figure 1). We found that all participants, regardless of geographical region, or antenatal care provider, moved through these three steps during the GDM counselling and screening process.

### 3.1 Informing oneself

The theme of ‘informing oneself’ reflected the first stage of the patient's care experience. This theme was underpinned by the factors that influenced the understanding of GDM, particularly pertaining to screening, prevention and management.

Accessing GDM resources was influenced by the availability of sources outside of direct antenatal care to improve participants' understanding and satisfaction with their GDM care experiences. External sources included websites, pamphlets, information sessions led by hospital staff, prenatal classes, independent research, conversations with family or friends and Facebook pregnancy groups. One person articulated:

I like it to be in a paper format, like a pamphlet, or some type of brochure. When I started going just to my OB/GYN, they would say, ‘here's some additional resources that you can read more about it’. That would have been really helpful with the gestational diabetes as well. (P2, FP)

Positive experiences with GDM information provision were described as factors that strengthened participants' knowledge acquisition, reduced pregnancy anxiety, and enabled participants to develop positive lifestyle habits through diet management and exercise. Negative experiences associated with a perceived lack of GDM information provision included feelings of frustration due to limited knowledge acquisition, as well as uncertainty in dietary and lifestyle modifications before and between testing:

I remember I started to change my diet, but then I wondered, is this going to impact the [GDM] test? If I get negative, would I go back to my old diet, being that I wasn't sure whether I should make the changes before, or would I have to wait until after the test? (P3, MW)

Participants' relationship with their antenatal care provider was an important consideration in obtaining GDM information. Some participants felt that the counselling they received was thorough, whereas others felt it was minimal. In general, more comprehensive counselling among patients in midwifery care was noted.

A range of discussion topics covered during counselling was reported and included: adverse outcomes, screening options such as the OGTT or the OGCT, risk factors associated with GDM and logistical considerations of the screening procedure such as the timing of fasting if required, and the type of sugar beverage administered.

Most participants, regardless of the care provider, reported feeling that they received sufficient information and were able to ask questions as needed. Many participants expressed appreciation for a provider who was accessible, supportive, demonstrated a calm demeanour and listened attentively. However, many reported that they would have liked to receive more counselling on specific topics, including prevention and management of GDM, signs and symptoms to be aware of, customized diet recommendations, and how to best prepare for the testing procedure:

I don't know if there's preventative measures that you can take to just prevent developing it. That would be helpful in terms of your diet or exercise. I don't feel like I got that information. (P3, MW)

Lack of communication emerged as a barrier to accessing information, including restrictive timelines and protocols for appointments, lack of follow‐up regarding the participant's GDM test results and inability to discuss health concerns due to restrictions in the scope of practice, or lack of educational training to provide patients with requested information.

### 3.2 Deciding

The theme of ‘deciding’ explored factors in the decision‐making process pertaining to GDM screening, including participant beliefs, values and healthcare autonomy. Prior beliefs about GDM and personal values regarding knowledge acquisition were explored; many participants viewed knowledge as a tool to prevent potential medical complications in pregnancy.

Almost all participants expressed pro‐universal GDM screening value statements. Beliefs that informed this included the following: (a) GDM is largely an asymptomatic condition, (b) GDM can affect anyone, regardless of risk profile, (c) screening is a minimally invasive and low‐risk procedure (the benefits outweigh the harms) and (d) screening improves one's awareness and motivation for the health of themselves and their developing baby:

From what I've seen, it's pretty random. I've seen people who are quite thin and healthy, people who aren't so healthy. Because of the effects it can have on the baby, I think it's important to be screened, because you don't necessarily know until it might be a bit late, and be causing significant effects. (P2, OB)

The degree to which participants were involved in the decision‐making process was particularly influential within this stage. Decisions included whether or not to be screened for GDM; choice of screening parameters such as the OGTT or OGCT; and the gestational time period to complete screening.

Being provided with an agency to make decisions was articulated as being very important for participants. For those who received care from a midwife, most reported that GDM screening was presented as optional and that they were able to make an informed decision based on the information provided. This was summarized best by one client, who stated:

With the midwife, she definitely made everything an option because she just wanted me to have more of an informed choice. So she told me ‘these are the tests that we highly recommend’, but really it was always up to me whether or not I wanted to do a test. (P9, MW)

For those who were not presented with GDM screening as ‘optional’ they described being instructed to be screened for GDM as it was simply ‘the thing to do’. One participant described this clearly when they stated, ‘I don't think it was presented to me as an option. It was presented to me as everyone gets screened, so I should get screened’ (P5, OB).

Furthermore, when asked about perceptions of the differences in counselling practices between antenatal care providers, many expressed the general belief that midwives have more time to provide comprehensive counselling, offering more opportunities for the client to make an informed decision:

OBs and FPs have such little time to sit and discuss things. Based on my experience of how it's gone in the past, I feel like there wouldn't be as much discussion and more just ‘you need to do this screen, here is the [requisition], go and do it’. (P10, MW)

Attitudes and motivation for engaging with health services were factors that influenced the level of importance that participants placed on healthcare autonomy. Some participants placed a significant emphasis on personal autonomy in their healthcare decisions. The participants that expressed the importance of making autonomous decisions were largely clients of midwives:

I think it's important for us that we are provided with information and that we are able to make the decision. We are supposed to live in a society where we are not forced to do things that we are not comfortable with. I think by having a midwife and them always making sure that they are informing my decisions, it's an awesome thing and obviously very empowering knowing that you are able to make these decisions on your own. (P12, MW)

Receptivity to screening was influenced by the risk factors that participants presented with and the extent to which they expressed concerns about its impact on the health of themselves and their babies. Many participants for whom screening was presented as optional ultimately expressed their receptivity to being tested given their risk factors:

[Screening] was available to me, and my OB thought it was a good idea based on the fact that I am older, I am overweight … and obviously with my family history of type 2. (P7, OB)

### 3.3 Screening

The last theme was related to experiences of completing the GDM screening test. Factors influencing the participant's access to testing were key determinants in their satisfaction with the screening process. Logistical barriers included challenges in obtaining childcare, inconvenient location of the laboratory, difficulty in scheduling time off work and transportation challenges. Individual reactions and experiences that presented barriers included distaste or aversion for the sugar beverage administered, difficulty coordinating fasting times before the test, emesis or nausea and discomfort with in‐person assessment during the COVID‐19 pandemic.

Factors that enabled screening were the absence of financial cost, ease of coordinating fasting times and taking time off work, the ability to attend the laboratory in a convenient location and on the weekend, a supportive partner that could transport the participant to and from their appointment, available childcare and minimal physical discomforts such as nausea.

Variations in screening practices included gestational timing, the type of testing approach recommended, and the locations where screening was offered. A few participants received earlier screening in their pregnancy due to the presence of risk factors: ‘My baby was trending quite large for my third pregnancy, so I did get screened earlier … I think given my BMI and whatnot … they just wanted to check and make sure that I didn't have it’ (P1, OB).

While most participants were screened within the recommended window of 24 to 28 weeks of gestation, a few individuals reported screening between 20 and 24 weeks gestation. No participants reported screening past 28 weeks. Of the 28 participants interviewed, 23 received the nonfasting 50 g OGCT; less than half required the follow‐up 75 g OGTT. The remaining five received the one step, fasting 75 g OGTT. Participants from rural areas experienced more limitations in lab capacities and screening times compared to cities.

## 4 DISCUSSION

This study explored the experiences of GDM counselling and screening from the perspectives of patients who received antenatal care from either a midwife, family physician or obstetrician in Hamilton, Sudbury or Ottawa, Ontario. The goal of this paper was to provide a qualitative analysis to explore the impact of variations in screening guidelines and changing patient population trends related to GDM counselling and screening practices.

Our findings highlight the progression of an individual's experience engaging with GDM health services through three stages: ‘informing oneself’, ‘deciding’ and ‘screening’. The findings within the stage of ‘informing oneself’ aligned with literature that supports the importance of comprehensive and personalized care provision according to the lived experiences and preferences of the individual.^14^, ^29^, ^30^ For example, much of the literature that explored patient perceptions GDM diagnosis highlighted feelings of self‐blame, failure, confusion and anxiety.^14^, ^29^, ^30^ These negative feelings were largely attributable to a lack of communication with their care provider, self‐perceptions of risk factors and a lack of information regarding adverse outcomes.^14^, ^29^, ^30^ Our participants also expressed how lack of communication, support or information provision impacted their experience.

Our findings highlight how autonomy and empowerment were tools for facilitating screening uptake and changes in health‐seeking behaviours.^31^, ^32^ For many participants, the autonomy that they had in decision‐making reflected their confidence in, or motivation for complying with, their care provider's recommendations. For example, many midwifery clients expressed strong motivation for being an active participant in the decision‐making process. These participants were more likely to value informed choice approaches, and were generally more expressive about their healthcare desires than those who received care from a physician. Instead, those receiving physician‐led care expressed enthusiasm to comply with their providers recommendations if it meant protecting the health of their baby.

We also found that factors such as reactions to the screening test and logistical considerations in accessing laboratory services, at the individual, organizational and health systems levels influenced participants' experiences. Barriers to obtaining screening reported in the literature were consistent with those expressed by participants in our study, including time restraints, inconvenient locations and transportation challenges.^17^, ^33^

In alignment with the literature, our findings indicated that there is a need for GDM care to be provided in a manner that is comprehensible, personalized and accessible, to best accommodate the lifestyle choices of diverse patient populations.^30^, ^34^ In particular, our study highlighted the importance of knowledge sharing as a facilitator in the decision‐making process.^14^, ^29^, ^30^, ^35^ Knowledge sharing is a reciprocal process that promotes patient empowerment, and encourages humility of the provider to foster a relationship built on mutual respect and rapport.^35^

Given the evolution of screening guidelines in recent years, care providers have had to regularly integrate these changes into their clinical practice. We had hypothesized that patients would be aware of and possibly confused by the variations in screening guidelines over time and this would be reflected in patient data. However, the findings showed the minimal impact of screening guidelines inconsistencies on the experiences of participants. Instead, logistical challenges, accessibility of the screening and personal preferences arose as primary influences on the participants' experiences.

One of the aims of this study was to explore the differences, if any, in the GDM counselling and screening practices of antenatal care providers across professions and across geographic regions within Ontario. Our findings revealed that while there were considerable differences in the participants' counselling experiences across care provider groups, there were very few differences across geographical regions. For example, those who received care from a midwife offered similar sentiments regardless of their geography, highlighting consistencies in professional philosophies across geographic regions. However, we acknowledge the restrictions of our sample to three geographic regions, which may not reflect the spectrum of individual and professional philosophies across the country.

The interpretation of access was also important to consider within the context of our study. Access can be conceptualized by reciprocal interaction between health structures and the ‘consumer’.^36^ It encompasses both accessibilities of providers, organizations, institutions and health systems to provide services, as well as the abilities of the consumer to receive such services, such as the ability to perceive, seek, reach, pay for and engage with health services.^36^, ^37^ As highlighted in our findings, information provision was a key factor in subsequent decision‐making and GDM screening uptake. However, to adequately interpret our findings, we must consider the multitude of agents that impact an individual's access to healthcare.^36^, ^37^

Lastly, the authors acknowledged that patient self‐selection of care providers was a key consideration in the findings. Those who selected to have midwifery‐led care may be inherently different that those who selected physician‐led care, particularly with respect to desired autonomy during decision‐making.^38^

Our study was unique in that it was one of the first to explore the experiences of patients seeking services pertaining to GDM counselling and screening, and compare across antenatal healthcare services, in a Canadian context. Furthermore, this study uniquely highlighted the direct impact that policy‐level guidelines have on patients and providers. Given the lack of qualitative evidence on this topic, the findings from this study provide valuable insight into what factors patients are most impacted by when seeking GDM counselling and screening.

Strengths of our study included the multidisciplinary nature of our team and our recruitment approaches to maximize participant variation to reflect the diversity that exists within the greater Ontario population, and to explore the range of social, cultural, economic and environmental factors that contribute to the experiences of health‐seeking patients. This enabled a range of perspectives that formed the basis through which comparisons of clinical practices across health sectors could be made.^39^

A limitation of our study was potential selection bias, given that those who volunteered to participate may be more willing to do so based on their personal beliefs about the topic. Furthermore, demographic characteristics were not obtained from five participants due to data collection documentation errors, presenting another source of bias. Additionally, our sample size reflected a lack of ethnic diversity, consisting of majority Caucasian identifying participants, with minimal to no participants from other ethnic groups. This may have further contributed to selection bias, with other populations not being well represented in our data. The COVID‐19 pandemic may have also presented possible selection bias which may have impacted recruitment for this study given the uncertainty in restrictions and research protocols. Also, during this time emergency alternatives to GDM screening protocols were published and may have introduced another variation in care.^40^ Lastly, language was also a limitation as interviews were only conducted in English.^41^

## 5 CONCLUSION

Our findings indicate that patients engage in GDM counselling and screening with a motivated mindset to protect the health of their babies. During the process of informing/deciding/screening, the informing stage and knowledge acquisition were crucial steps for facilitating decision‐making and screening uptake. However, there were differences in the perceptions of the comprehensiveness of GDM counselling between antenatal care providers. The desire for patients to be active participants in decision‐making is a reflection of their selection of care providers. Useful next steps to improve the patient experience include training for health professionals, and the creation of patient information resources that are adapted to the needs, preferences and lifestyles of patients, as well as a greater emphasis on information provision to facilitate patient autonomy.

## CONFLICT OF INTEREST

The authors declare no conflict of interest.

## ETHICS STATEMENT

Ethical approval was obtained from the Hamilton Integrated Research Ethics Board (HiREB Project: 7916). All participants provided consent before participation in the study.

## Supporting information

Supporting information.

## Data Availability

The data sets generated and/or analysed during the current study are not publicly available due to the lack of consent from the study participants to share the data publicly but are available from the corresponding author at a reasonable request.
