# Supplementary material for: Exploring patients' perspectives of gestational diabetes mellitus screening and counselling in Ontario: A grounded theory study
Source: Health Expect. 2023 Jan 18;26(2):827–35. doi: 10.1111/hex.13708 (PMC10010101; doi:10.1111/hex.13708)
Supplement: Supplementary file 1 — Supporting information. [file HEX-26--s001.docx]

# **Supporting Information (S1): Interview Guide – Pregnant Women**

**Demographic Questions**

1) **Type of health care provider:**

- Midwife
- Family
- Physician Obstetrician

2) **Age:**

- 15-24
- 25-34
- 35-44
- 45-54
- 55-64

3) **Ethnic or cultural origin:**

- First Nations
- South Asian
- East Asian (Chinese, Filipino, Japanese, Korean)
- Black (African, Haitian, Jamaican, Somali)
- White
- Other

4) **Highest level of education:**

- High School
- Bachelors
- Graduate/Professional Doctorate

5) **Parity:**

- Nulliparous (first pregnancy?)
- Multiparous (any past pregnancies?)

**Interview Questions**

1. Why are you interested in participating in this study?

2) Did you receive any counselling on GDM during your pregnancy?

If yes…

a) Can you describe what topics were covered during that counselling?

Possible probes: what is GDM, risk factors for GDM, adverse outcomes related to GDM, prevention of GDM, screening for GDM

b) When did you receive this counselling during your pregnancy?

c) Do you think women should be screened for GDM?

3) Were you given the option to get screened for GDM?

a) At what point in your pregnancy did you get screened?

b) How were you screened?

c) What was your experience with screening? Were there any challenges?

If you screened positive for GDM what resources were available or offered to you? (e.g., dietician, medication)

4) Did you feel that you were provided with sufficient information on GDM in pregnancy?

a) Were you able to ask questions to your health care provider about GDM?

b) Were your questions adequately answered?

5) What additional knowledge would you have liked to have about GDM in pregnancy?
